# Metastatic seeding of glioblastoma along image-guided biopsy tract with successful treatment with re-irradiation: a case report

**DOI:** 10.3332/ecancer.2012.264

**Published:** 2012-08-15

**Authors:** A Pascoe, R Vijayan, M Kumar, G Dow

**Affiliations:** 1 Department of Oncology, Royal Derby Hospital, Uttoxeter Rd, Derby, DE22 3NE, UK; 2 Department of Oncology, Colchester Hospital University NHS Foundation Trust, Lexdon Rd, Colchester, Essex, CO33NB, UK; 3 Department of Neurosurgery, Queen’s Medical Centre, Nottingham, NG7 2UH, UK

**Keywords:** *image-guided brain biopsy tract*, *glioblastoma*, *metastatic seeding*, *re-irradiation*

## Abstract

Metastatic seeding along image-guided brain biopsy tracts is an uncommon phenomenon. We report a case of meningeal glioblastoma (GBM) metastasis at the site of a previous image-guided biopsy site, which developed 14 months after completing concurrent chemo-radiotherapy. This is the first reported case of successful treatment of an intracranial GBM metastasis with resection followed by radiotherapy.

## Introduction

Image-guided brain biopsy is now commonly utilised for diagnosis of brain neoplasms. Metastatic seeding along the biopsy tract is a rare but recognised complication in a range of different pathologies and more recently in glioblastoma (GBM). We present the first reported case of successful resection and radiotherapy treatment of a meningeal GBM metastasis.

## Case report

**Figure 1:**
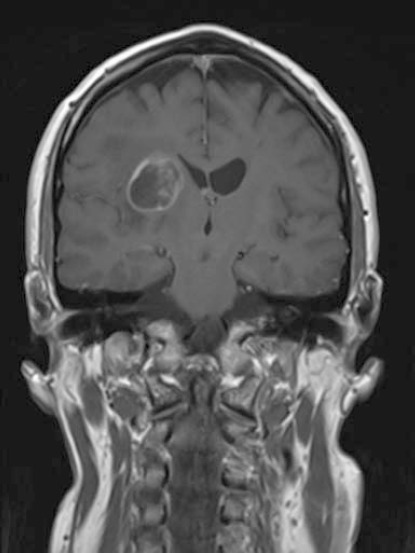
MRI (T1-weighted with gadoliunium) at presentation in April 2008.

A 52-year-old right-handed gentleman was presented in April 2008 with a mild left hemiparesis and slurred speech. An MRI of the brain showed a 25 × 24 mm mass lesion in the deep right hemisphere area adjacent to the thalamus, which was well defined and of low-signal intensity on T1-weighted images and high-signal intensity on T2 with some low-signal septations within it. There was significant perilesional oedema, and following gadolinium there was ring enhancement and some enhancement within the septations. Appearances were therefore in keeping with a high-grade glioma (Figure 1).

The patient went on to have a CT-guided biopsy (Medtronic Stealth™), and the histology confirmed GBM multiforme, WHO grade 4. He therefore went on to be treated with radical radiotherapy to a dose of 60 Gy in 30# over six weeks with concurrent Temozolamide chemotherapy. An MRI scan following this showed stable disease, so he proceeded with adjuvant Temozolamide chemotherapy starting at 150 mg/m^2^ and escalating to 200 mg/m^2^ from cycle 2. An interval MRI scan in November 2008 showed slight but definite partial response but significant residual tumour volume, so he continued to a total of six cycles of Temozolamide which completed in February 2009. This was then followed by a period of observation, and a further MRI in May 2009 showed a marked reduction in the size of the tumour, with >50% reduction in tumour volume and only a very mild degree of peripheral enhancement.

**Figure 2:**
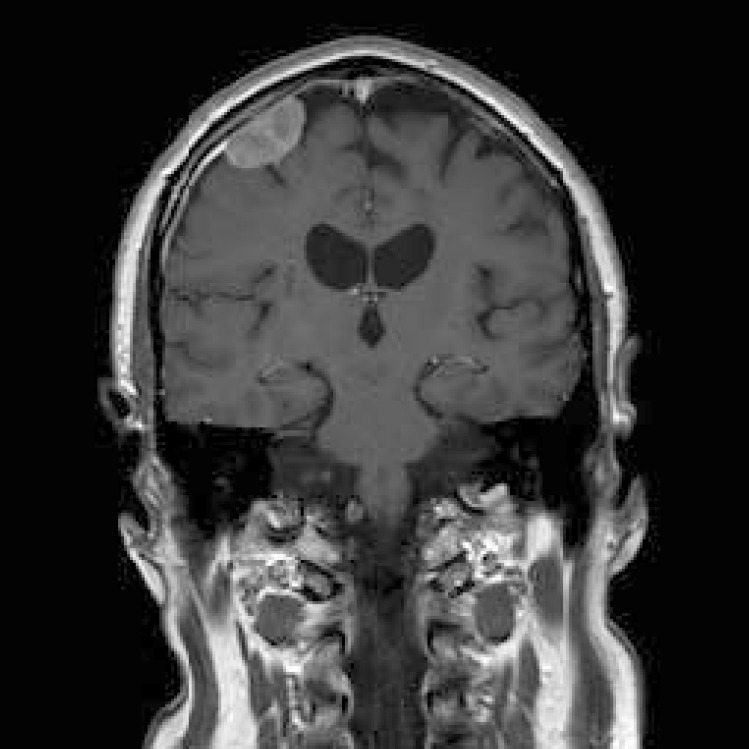
MRI (T1-weighted with gadolinium) at detection of seeded metastasis September 2009.

The patient remained well until September 2009, when he was presented with a visual aura, and an urgent MRI scan showed that the primary tumour continued to decrease in size, but that there was an additional 27-mm enhancing extra axial lesion in the right frontal lobe, appearing to arise from the dura just adjacent to the burr hole site from the previous biopsy (Figure 2). This was discussed in the regional neuro-oncology MDT, and the differential diagnosis was thought to be between meningioma and metastatic GBM. Due to the rapid growth of this in the 4 months since the last MRI, GBM was thought to be more likely and he therefore underwent an image-guided craniotomy and resection of the nodule, with histology confirming GBM.

On reviewing the patients’ previous radiotherapy plans, it was noted that the new nodule was 7 mm from the previous radiotherapy field, and further treatment with radiotherapy would therefore require a significant overlap in radiotherapy fields, although no critical structures such as the optic chiasm were close to this region of overlap. Following discussion with other centres regionally and nationally, a consensus was reached to retreat with radiotherapy, and the patient was consented for a 10% risk of brain necrosis in the region of overlap. He had further radiotherapy to a dose of 30 Gy in 6# over two weeks (three times a week) in December 2009, and a follow-up MRI scan in May 2010 showed no residual enhancement in the original tumour, and the meningeal deposit was no longer visible.

The patient continued to remain well until January 2011, when a routine MRI scan showed tumour recurrence and he commenced palliative chemotherapy with PCV; unfortunately despite an initial good response to PCV he began to deteriorate clinically, and chemotherapy was stopped in July 2011.

## Discussion

Metastases of GBM multiforme are uncommon and when they do occur, they are often related to surgical procedures such as brain biopsy and ventriculo-peritoneal shunt insertion. Frameless stereotactic biopsy with image guidance using neuro-navigation systems are now commonly used for biopsy of brain tumours with high-diagnostic yield and low morbidity. Possible complications described include haemorrhage and infection or neurological deficit. Cases have been reported of seeding along biopsy sites in brain neoplasms including: brain metastases, craniopharyngioma, and anaplastic astrocytoma. There are three previously reported cases [1–3] of intracranial seeding of GBM multiforme along biopsy tract detected by radiological follow up; a further case [4] was also reported of an epidural metastatic deposit at biopsy site on post-mortem.

The first case reported [1] was of GBM treated with stereotactic radiotherapy, and it was therefore hypothesised that the seeding along the biopsy tract occurred as the needle tract was not within the radiotherapy field. A second case [2] was treated with external beam radiotherapy, and the needle biopsy tract was within the radiotherapy field this time; potential hypotheses at that time were that GBM can be radio-resistant and that further boost to biopsy tract may be needed, or that radiotherapy needed to be started more quickly following biopsy. However, in both these cases, the secondary tumours occurred within three months of the radiotherapy. Our case is unusual as our patient was not presented with a metastatic deposit until 14 months after completing his radiotherapy. Of interest, he received concurrent chemo-radiotherapy followed by adjuvant chemotherapy, which, since the Stupp trial [5], is the standard of care for patients with good performance status and GBM. The third case [3] previously presented was also treated with concurrent chemo-radiotherapy, and metastatic seeding was detected 8 months after treatment. This along with our case suggests that the addition of chemotherapy alongside radiotherapy may delay the presentation of metastatic seeding.

Our case is the first reported of treatment of an intracranial GBM metastasis with further radiotherapy after resection. Re-treatment of our patient with radiotherapy did involve an area of overlap with the original radiotherapy field but was successfully completed and the patient had 13 months before further disease progression. A review of re-irradiation tolerance of the brain with respect to treatment of gliomas [6] found that normal brain tissue necrosis occurred with normalised total doses (NTD) of >100 Gy and concluded that re-irradiation was a feasible option for palliative treatment of recurrent high-grade glioma.

There have been an increasing number of reports of patients with both intracranial and distant metastases in GBM multiforme in recent years. Although this is still a very uncommon occurrence, it may be that improvements in survival since the Stupp trial [5] will also lead to an increase in the development of metastatic disease. Intracranial metastatic seeding of GBM is an uncommon but recognised complication of image-guided brain biopsy. Treatment with concurrent chemo-radiotherapy may delay the development of metastatic deposits along the biopsy tract; however, if patients continue to have good performance status and control of the primary tumour, then treatment of the metastasis with further radiotherapy should be considered.
